# Small Extracellular Vesicle-Associated microRNA in Cancer: Biology and Applications in Translational Research and Precision Oncology

**DOI:** 10.3390/cancers18121903

**Published:** 2026-06-11

**Authors:** Konstantinos Karamouzis, Ioannis Kollias, Maria Trapali, Maria Papatsirou, Maria Gavriatopoulou, Ioannis Ntanasis-Stathopoulos

**Affiliations:** 1 Department of Clinical Therapeutics, School of Medicine, National and Kapodistrian University of Athens, 11528 Athens, Greece; kkaramouzis02@gmail.com (K.K.); gkollias286@yahoo.gr (I.K.); papatsir@biol.uoa.gr (M.P.); mgavria@med.uoa.gr (M.G.); 2 Laboratory of Chemistry, Biochemistry and Cosmetic Science, Department of Biomedical Medicine, University of West Attica, 12243 Egaleo, Greece; ymaria@uniwa.gr

**Keywords:** small extracellular vesicles (sEVs), exosomes, miRNA, extracellular vesicle-associated micro-RNAs (EV-miRNAs), cancer, liquid biopsy, diagnosis, prognosis, treatment, personalized medicine

## Abstract

Small extracellular vesicles (sEVs) are extracellular vesicles that mediate intercellular communication through the transfer of bioactive molecules, including proteins, lipids, and nucleic acids. Among their cargo, microRNAs (miRNAs) have emerged as critical regulators of gene expression with significant implications in cancer biology. Tumor-derived extracellular vesicle-associated microRNAs (EV-miRNAs) can reprogram recipient cells, promoting oncogenesis, metastasis, angiogenesis, and therapeutic resistance. This review provides a comprehensive overview of EV-miRNAs in cancer, examining their biogenesis, mechanisms of intercellular transfer, and functional roles in tumor progression. We discuss the clinical potential of EV-miRNAs as non-invasive biomarkers for cancer diagnosis and prognosis, as well as their emerging applications in targeted therapeutic strategies. Furthermore, we address current challenges related to isolation techniques, quantification methods, and standardization protocols that hinder clinical translation. Finally, we outline future directions for integrating EV-miRNA analysis into precision oncology frameworks and liquid biopsy platforms, highlighting opportunities to advance personalized cancer care.

## Simple Summary

Cells communicate with each other by releasing tiny vesicles called small extracellular vesicles (sEVs), which act as molecular messengers carrying a variety of biological molecules between cells. Among these molecules, microRNAs, which are short non-coding RNA molecules, play a key role in regulating whether genes are switched on or off in the recipient cells. In cancer, this communication system can be hijacked: tumor cells release sEVs loaded with specific microRNAs that reshape the surrounding environment to support cancer growth, metastasis, angiogenesis, and resistance to treatment. This review explores how extracellular vesicle-associated microRNAs (EV-miRNAs) contribute to cancer development and progression and examines their potential use as minimally invasive markers for early diagnosis and disease monitoring through blood-based tests. Potential therapeutic strategies targeting these molecules are also discussed, with the aim of advancing their integration into personalized cancer care.

## 1. Introduction

Small extracellular vesicles (sEVs) play a central role in intercellular communication by serving as mediators and vehicles for the transfer of diverse biomolecules—proteins, lipids, DNA, mRNA, and non-coding RNAs (such as microRNAs)—between cells. sEVs can interact with cell-surface receptors, triggering signaling cascades in recipient cells, or be internalized, releasing their molecular cargo to directly modulate gene expression and cellular functions. This mode of communication is crucial for maintaining physiological homeostasis, but in pathological contexts such as cancer, alterations in sEV cargo composition can promote malignant transformation and tumor progression, and modulate the behavior of neighboring or distant cells. Among the molecular cargo transported by sEVs, microRNAs (miRNAs) have emerged as key regulators of cancer biology. Dysregulation of certain miRNAs can contribute to oncogenesis by promoting tumor growth, metastasis, angiogenesis, and resistance to therapy. Cancer cells often release extracellular vesicle-associated microRNAs (EV-miRNAs) that alter gene expression in neighboring or distant cells, promoting an environment conducive to tumor progression. This review aims to provide an overview of the role of EV-miRNAs in cancer. Specifically, we examine their biology, mechanisms of action, and clinical applications, including their use as biomarkers for diagnosis and prognosis, as well as their potential as targets of therapeutic strategies. Challenges in the analysis and standardization of EV-miRNAs are also addressed, and future directions for their integration into precision oncology and liquid biopsy platforms are discussed.

### Review Methodology

To ensure a rigorous and comprehensive synthesis of the literature, a systematic search was conducted across the PubMed, Scopus, and ScienceDirect databases, complemented by an assessment of ongoing and completed clinical studies registered in ClinicalTrials.gov. The search strategy was designed to capture the intersection of vesicle biology and clinical oncology using keywords such as “small extracellular vesicles (sEVs),” “exosomes,” “miRNA,” “EV-miRNAs,” and “exomiR.” The scope was specifically bounded to studies investigating these vesicle-associated mediators in the context of cancer pathogenesis, focusing on their role in liquid biopsy, diagnosis, prognosis, and personalized medicine.

Only peer-reviewed original research articles and authoritative review papers published in English were included, with a primary focus on the past decade to capture the most recent technological and clinical advancements. A narrative synthesis approach was used to integrate diverse findings—ranging from basic mechanisms of biogenesis and signaling mechanisms to the challenges of standardization in quantification—into a cohesive framework for precision oncology applications.

## 2. sEV Biogenesis and miRNA Loading Mechanisms

### 2.1. Overview of sEV Biogenesis and Secretion Pathways

Extracellular vesicles (EVs) are cell-derived, membrane-enclosed particles released by virtually all cell types into the extracellular space. They serve as intercellular communication vehicles by transferring a diverse cargo—including proteins, lipids, nucleic acids, and metabolites—to recipient cells in both local and distant tissues, thereby regulating a wide range of physiological and pathological processes. EVs are broadly classified according to their biogenesis, size, and presumed cellular origin into three classical subtypes—exosomes, microvesicles, and apoptotic bodies. While the classical subtypes are mutually exclusive based on their distinct mechanisms of formation, the experimental discrimination of EV subpopulations remains technically challenging and requires advanced isolation techniques [1].

Exosomes originate from the endosomal pathway and are generated through the maturation of multivesicular bodies (MVBs), which contain numerous intraluminal vesicles (ILVs). The subsequent fusion of MVBs with the plasma membrane results in the extracellular release of these ILVs as exosomes [2,3].

ILVs are generated by inward budding of the endosomal limiting membrane. ILV formation can occur through two principal mechanisms: one dependent on the endosomal sorting complex required for transport (ESCRT complex) and one independent of it. The ESCRT machinery comprises multiple protein complexes (ESCRT-0, -I, -II, -III, and Vps4). Initially, ESCRT-0 recognizes ubiquitinated cargo proteins on the membrane and binds to them. This sequentially activates the remaining components of the complex, leading to the formation of membrane protrusions within the endosomes. Membrane scission is then mediated by ESCRT-III and the Vps4–Vta1 complex, resulting in the formation of ILVs. The accumulation of ILVs inside an endosome leads to the formation of an MVB [2].

**Figure 1:**
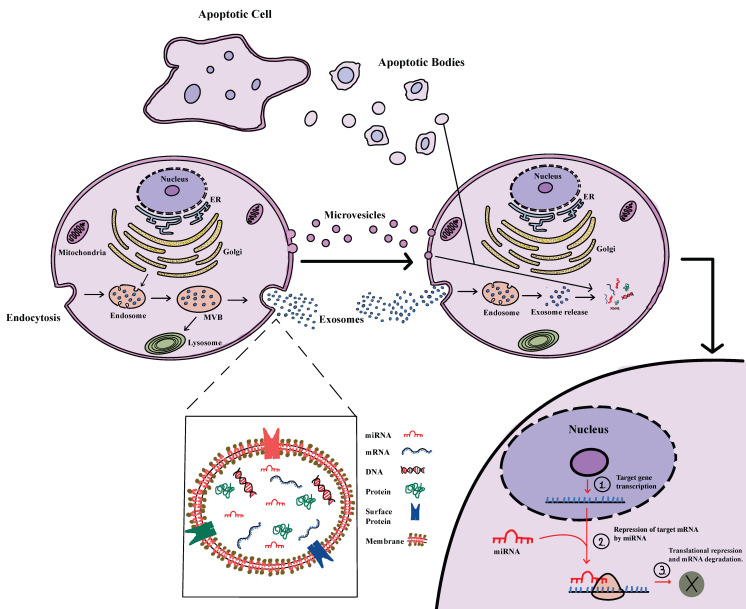
Biogenesis, secretion, and molecular composition of extracellular vesicles (EVs) and their role in intercellular communication. The figure illustrates the three main subpopulations of EVs: (i) exosomes, originating from the endosomal pathway via multivesicular bodies (MVBs); (ii) microvesicles, formed by direct budding of the plasma membrane; and (iii) apoptotic bodies, released during programmed cell death. The magnified view highlights the complex cargo of sEVs, including miRNAs, mRNAs, DNA, proteins and surface proteins (depicted in different colors to represent distinct surface protein subtypes). Upon uptake by recipient cells, exosomal miRNAs mediate post-transcriptional gene silencing by targeting specific mRNAs, leading to translational repression and mRNA degradation (represented by the circled “X” symbol).

MVBs containing ILVs can fuse with the plasma membrane via the cytoskeletal network and microtubules, releasing exosomes. Alternatively, they are directed towards degradation by fusion with lysosomes or autophagosomes, as depicted in Figure 1 [2].

### 2.2. Mechanisms of Selective miRNA Sorting into sEVs

The RNA content of exosomes can be dependent only on the total cellular miRNA content and independent of specific mechanisms, a process called passive miRNA loading. Alternatively, active miRNA loading into exosomes is mediated by RNA-binding proteins (RBPs), which selectively bind miRNAs and transport them to ILVs, acting as adapters between various RNA entities and the assembly complexes of the exosome [4].

Mechanisms related to specific sequences in the RNA molecule have also been observed. More specifically, during selective loading—a category of active loading—specific miRNA species are selected based on specific sequence motifs called EXOmotifs. In contrast, miRNAs containing CELLmotifs tend to be retained in the cell of origin. This mechanism acts independently of miRNA abundance in the cell of origin [5].

Active miRNA loading also encompasses mechanisms related to post-translational modifications of RBPs. The hnRNPA2B1 protein is an example of a protein that directs miRNA towards secretion via exosomes upon SUMOylation. LC3 conjugation has also been associated with secretory small RNA cargo loading in the context of autophagy [6].

## 3. Functional Roles of EV-miRNAs in Cancer Biology

### 3.1. Tumor–Tumor Communication

EV-miRNAs can support processes that favor cancer cells, such as proliferation, invasion, and epithelial–mesenchymal transition (EMT). Such vesicles are often secreted by cancer cells or by cells in the tumor microenvironment.

Accumulating evidence supports the notion that cancer cells engage in crosstalk via sEV-miRNAs that promote processes such as growth, invasion, and proliferation. Tumor-derived miR-7641 has been reported to induce tumor growth and enhance the metastatic potential of cells in breast cancer (BrCa). miR-21 regulates multiple oncogenic processes in early-stage lung adenocarcinoma (LUAD), including cell-cycle progression, DNA-damage repair, and EMT [7]. Its overexpression is also significantly associated with poorer survival outcomes in patient cohorts [8,9]. Another notable example of the effect of EV-miRNAs on tumor progression is miR-155-5p, which acts on recipient cancer cells by accelerating growth through the activation of the JAK2–STAT3/NF-κB pathway [10]. In osteosarcoma (OS), sEV-derived miR-19a-3p promotes osteoclast differentiation and activity through PTEN inhibition, which leads to increased AKT phosphorylation. The resulting activation of AKT further stimulates osteoclast differentiation and function, contributing to bone destruction and promoting OS metastasis [11].

### 3.2. Angiogenesis and Premetastatic Niche Formation

It is already known that in the development of cancer, oxygen and nutrient supply are critical for tumor growth. More specifically, the rapid proliferation of cells and the increase in their metabolic demands heighten the need for larger amounts of oxygen and nutrients, which are supplied through the bloodstream. Angiogenesis is therefore a process of critical importance for tumor progression, as it provides the nutrients and oxygen required for cancer cell growth and proliferation, while also facilitating metastasis to distant tissues and organs [12].

One of the stimulating mechanisms that trigger angiogenesis is the secretion of sEVs by solid tumors under hypoxic conditions. These tumor-derived sEVs contain miRNAs, such as miR-9, which convert fibroblasts into cancer-associated fibroblasts (CAFs), thereby enhancing the production of angiogenic growth factors such as VEGF. In addition, miRNAs such as the miR-17-92 cluster induce endothelial migration and vascular tube formation [13,14,15,16].

miRNAs such as miR-105 and miR-181c have been found to participate in brain metastasis as they are taken up by the endothelial cells of the blood–brain barrier, promoting its disruption by modifying the cytoskeleton [17,18,19].

High levels of miR-105, when transported extracellularly and taken up by pulmonary microvascular endothelial cells, lead to a significant reduction in ZO-1. This process increases vascular permeability, which in turn facilitates the colonization of the lungs by metastatic cancer cells. Thus, in addition to the angiogenic capacity of miRNAs in cancer, they can create a premetastatic environment that favors the growth and formation of secondary metastatic foci [20].

Moreover, miR-210 has been found to be enriched in sEVs in high concentrations under hypoxic conditions and is released by cancer cells to induce the angiogenic activity of endothelial cells [21].

Multiple myeloma cells cultured under hypoxic conditions produce sEVs containing large amounts of miR-135b, which directly inhibits FIH-1, an inhibitor of HIF-1, thereby promoting endothelial tube formation [22,23,24].

In oral squamous cell carcinoma (OSCC), cancer cells can secrete sEVs containing miR-210-3p. This miRNA promotes tumor growth by increasing microvessel density as it reduces the expression of ephrin A3 by stimulating the PI3K/Akt pro-angiogenic pathway [25]. In nasopharyngeal carcinoma (NPC), cancer cells have a high metastatic capacity, and extracellular miR-23a has been found to play an important role in angiogenesis, which is key to metastasis in this type of cancer. miR-23a binds to the untranslated region (3′-UTR) of TSGA10, an angiogenesis suppressor, reducing its expression, leading to angiogenesis and increased metastasis of nasopharyngeal carcinoma cells [26].

miR-155 has been found to be transported by sEVs that target endothelial cells, promoting angiogenesis in gastric cancer. miR-17-5p derived from nasopharyngeal carcinoma exosomes targets BAMBI, a pseudoreceptor of TGF-β, promoting angiogenesis, proliferation, and cell migration while regulating the AKT/VEGF-A signaling pathway. In ovarian cancer, increased levels of miR-205 expression were found within cancer-derived exosomes, leading to the metastasis of endothelial cells by promoting angiogenesis. In pancreatic cancer, cancer cell-derived sEV-derived miR-27a promotes angiogenesis and endothelial cell activation [27,28].

Beyond initiating angiogenic processes in carcinogenesis, miRNAs can induce tumor metastasis to other tissues and organs. Substantial evidence supports the involvement of miRNAs in conditioning the premetastatic niche. miRNAs such as miR-10b, which are contained within cancer sEVs, are secreted by breast cancer cells, inducing the invasiveness of non-malignant breast epithelial cells [29].

miR-25-3p has been identified in colon cancer-derived sEVs. This miRNA enters the surrounding epithelial cells, disrupting the integrity of their connections and thus promoting metastasis to the liver and lungs. Similarly, in breast cancer, miR-105 targets ZO-1 tight junctions, resulting in the destruction of blood vessels and increased vascular permeability. This increases the likelihood of metastasis to the brain or lungs. This miRNA was also found to be elevated in the blood of patients with distant metastasis or metastasis to the lymph nodes.

In another study, miR-181c derived from metastatic breast cancer cells in the brain was found to cause abnormal localization of claudin-5, occludin, ZO-1, N-cadherin, and actin through the transfer of miR-181c to endothelial cells of the blood–brain barrier, resulting in the disruption of intercellular contact. These results are directly associated with elevated circulating levels of miR-181c in the blood of breast cancer patients with secondary brain metastases. Similarly, extracellular miR-103a-3p compromises the integrity of endothelial cell junctions, promoting tumor metastasis by targeting VE-Cad, p120, and ZO-1.

In oral cancer, highly metastatic tumor cells have been shown to release sEVs containing miR-342-3p and miR-1246, which can be taken up by less metastatic cancer cells, resulting in increased motility and invasiveness [30,31]. miR-223, guided by IL-4 in target cells (breast cancer cells), acts through the MEF2C-β-catenin pathway, enhancing the migration and invasion of breast cancer cells. In patients with advanced esophageal cancer, miR-21 levels were elevated. miR-21 was associated with lymph node metastases and inflammatory reactions, promoting metastasis [32].

### 3.3. Tumor–Microenvironment Interactions

#### 3.3.1. Modulation of Cancer Cells by sEV Cargo Derived from the Tumor Microenvironment

The tumor microenvironment (TME) and the cells that comprise it also play a central role in disease development, progression, and treatment resistance. A key mechanism by which this is achieved is through the secretion of EV-miRNAs by TME cells, resulting in a bidirectional sEV crosstalk between tumor cells and cells of the microenvironment. A typical example is cancer-associated fibroblasts (CAFs), which modulate cancer cell behavior through multiple mechanisms [33].

For instance, CAF-Exo miR-196a promotes the development of cisplatin resistance in head and neck cancer cells by silencing cell-cycle-regulatory proteins [34,35]. In gastric cancer, miR-522 also promotes resistance to cisplatin [36]. In pancreatic cancer, CAF-Exo miR-106b reduces the expression of tumor protein 53-induced nuclear protein 1 (TP53INP1), subsequently inducing gemcitabine resistance [37]. Additionally, miR-210-3p derived from bone marrow stem cells (BMSCs) has been shown to activate the Wnt/β-Catenin pathway, a regulator of stemness, invasion, and proliferation in triple-negative breast cancer (TNBC) cells [38]. Conversely, miRNAs that negatively regulate PD-L1 act as tumor suppressors by inhibiting PD-1/PD-L1 interaction. In non-small cell lung cancer (NSCLC), miR-4458 and miR-34 have been shown to regulate PD-L1 by targeting STAT3 and p53, respectively [39].

#### 3.3.2. Cancer-Cell EV-miRNAs Modulate Immune Cells

Cancer cells modify their environment to benefit their survival and proliferation. This is achieved not only directly, through processes such as angiogenesis and the formation of the metastatic niche, but also through the reprogramming of immune cells inducing phenotypes that favor the survival of cancer cells. Cellular crosstalk via sEVs and EV-miRNAs has also been shown to play a key role in this process.

A noteworthy example is tumor-associated macrophages (TAMs), cells that, among other functions, suppress T-cell activity, thereby assisting in the immune evasion of cancer cells. Macrophages tend to develop a TAM phenotype after crosstalk with cancer cells. In the context of pancreatic cancer, it has been shown that miR-182-5p found in EV-enriched in exosomes leads to increased PD-L1 expression, which suppresses T-cell activity. Furthermore, miR-182-5p recipient TAMs show reduced expression of TLR-4, limiting related antitumor immune responses [40]. In colon cancer, it has been observed that sEV-associated miR-1246 modulates the TGF-β pathway in recipient macrophages, driving them towards a tumor-supportive phenotype [41,42].

**Figure 2:**
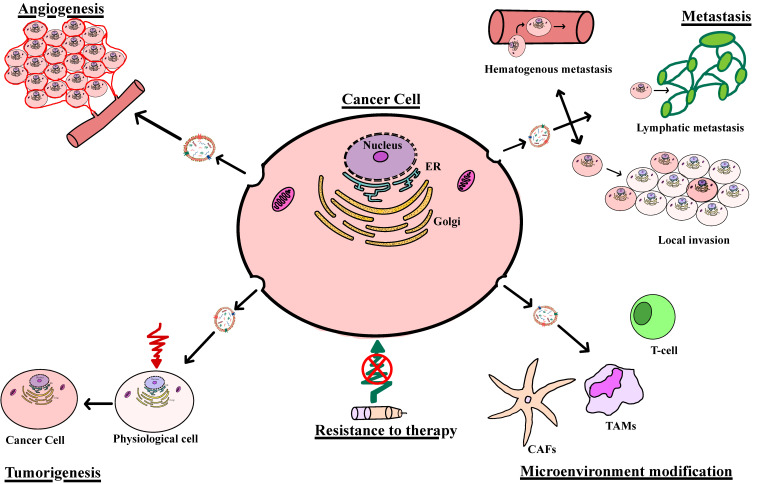
MicroRNAs (miRNAs) play a critical role in cancer biology, where their dysregulation contributes to tumor growth, metastasis, angiogenesis, and therapy resistance. Tumor-derived EV-miRNAs further modulate gene expression in local and distant cells, fostering a microenvironment that supports cancer progression. Cancer-associated fibroblasts (CAFs), tumor-associated macrophages (TAMs), and T cells actively contribute to the dysregulation of the tumor microenvironment through EV-miRNAs. CAF-derived EV-miRNAs promote tumor progression and therapy resistance, TAMs adopt an immunosuppressive phenotype that inhibits T-cell activity, and T cells undergo functional impairment, collectively supporting tumor growth and immune evasion.

In the case of multiple myeloma, sEV-associated miR-27b-3p displays different effects depending on the target cell. In cancer recipient cells, it causes the downregulation of FBXW7, leading to the stabilization of MYC and increased proliferation, whereas when taken up by CD3^+^ T cells via sEVs, it induces immunosuppressive phenotypes with CD28^−^CD57^+^ paired with reduced cytokine secretion [43]. The mechanisms described above are schematically illustrated in Figure 2.

## 4. Clinical Relevance of EV-miRNAs in Cancer

### 4.1. Diagnostic and Prognostic Biomarkers

Given the current understanding of EV-miRNAs and their role in intercellular communication, these molecules have emerged as valuable tools in cancer research. They contribute to a better understanding of the complex biology of cancer. They also hold significant translational potential. EV-miRNAs can support cancer diagnosis, disease monitoring, prognostic assessment, and therapeutic targeting.

miRNAs can be detected either as free-circulating molecules or enclosed in sEVs. The latter are considered to have greater clinical utility, as free-circulating RNA molecules are generally highly susceptible to hydrolysis and enzymatic degradation. Additionally, EV-miRNAs reflect the molecular profile of their cells of origin, providing valuable tumor-associated information through liquid biopsy [44,45,46].

Given the growing interest in EV-miRNAs as liquid biopsy biomarkers for early cancer detection and prognostic stratification, a growing body of clinical trials focusing on the utility of EV-miRNAs has emerged. In Table 1, we summarize the current clinical studies that utilize EV-miRNA detection in cancer patients based on the ClinicalTrials.gov database (see also Supplementary Table S1 for more details).

Sui et al. developed a panel of five overlapping miRNAs detectable both in circulation and sEVs as part of the multicenter DESTINEX study for the early detection of gastric cancer (GC). This signature of five circulating and extracellular miRNAs was derived from larger sets of circulating and extracellular miRNAs that were differentially expressed in tumor versus adjacent tissue from patients with GC. The diagnostic sensitivity and specificity of the panel (miR-21-3p, miR-21-5p, miR-215-5p, miR-27a-3p, and miR-95-3p) were exceptionally high in the validation cohort, underscoring the importance of detecting extracellular miRNAs through liquid biopsy for the early identification of patients with GC [47].

Another noteworthy example of the emerging role of EV-miRNAs in liquid biopsy and cancer biology is the recently published study by Choudhary et al., which identified a panel of five EV-miRNAs (hsa-miR-6803, hsa-miR-1180, hsa-miR-4728, hsa-miR-1915, and hsa-miR-940) that may provide valuable information for early diagnosis, prognosis, and assessment of stemness in TNBC [48].

### 4.2. Predictive Biomarkers and Therapeutic Monitoring

The ability of sEV cargo to provide information about the cells of origin—and thus information directly related to the tumor—can also be used to monitor disease progression and response to therapy, thereby aiding in outcome prediction and treatment monitoring.

In hepatocellular carcinoma (HCC), sEV-associated miR-92b and miR-215-5p have been shown to have predictive value for recurrence following curative therapy such as resection or ablation [40,41,42,43,44,45,46,47,48,49,50,51]. The simultaneous analysis of sEV cargo in combination also serves as a strong predictive tool. It has been observed that the combined detection of sEV-derived miR-150-3p and the NMT2 and PRDM1 mRNAs can be useful in predicting acute tumor response (AR) in locally advanced cervical cancer (LACC) receiving concurrent chemoradiotherapy (CCRT). The panel demonstrated a strong correlation with AR with R^2^ = 0.831 and *p*-value < 0.0001 [52].

Conversely, some miRNAs are associated with favorable disease outcomes by suppressing progression and thus they can be used as markers of a favorable prognosis and predictors of treatment response. The diversity of mechanisms in which ncRNAs are generally involved renders them promising candidate molecules for therapeutics, as will be discussed subsequently. An example of this favorable behavior is the inhibition of BRAFi resistance in melanoma by sEV-associated miR-7 [53].

### 4.3. Therapeutic Applications of miRNAs Enclosed in Exosomes

Exosomes have been studied for their therapeutic applications as drug delivery vehicles, as well as for the delivery of biomolecules that enhance antitumor activation of the immune system, such as cancer antigens, checkpoint inhibitors, and miRNAs with tumor-suppressing properties [54]. They can significantly contribute to cancer therapy due to their reduced toxicity. The therapeutic cargo is protected within sEVs, which enhances its stability and delivery. Moreover, sEVs exhibit improved targeting of cancer cells through their surface proteins, particularly when they are generated via the ESCRT pathway [55].

Chimeric exosomes are of particular interest, as they are engineered to express both tumor antigens and immune checkpoint inhibitors, thereby activating dendritic cells and T cells, achieving an enhanced immune response [56]. Another emerging entity of particular interest in cancer therapy research is hybrid exosomes. These vesicles result from the fusion of a laboratory-synthesized nanoparticle (primarily a liposome) with an exosome. Their significance lies in their potential for low immunogenicity and targeted drug delivery, resulting in improved efficacy and reduced toxicity [57].

miRNAs are of considerable relevance in cancer therapeutic interventions since, as described above, their context-dependent roles in disease development and progression have been clearly established. The so-called oncogenic miRNAs promote tumorigenesis, disease progression, and resistance to therapy by suppressing the expression of tumor-suppressor genes. Conversely, tumor-suppressive miRNAs inhibit the translation of oncogene mRNAs, thereby acting as tumor suppressors.

Accordingly, several oligonucleotide-based therapeutic agents have been developed to enhance the effect of tumor-suppressive miRNAs or suppress oncogenic miRNAs.

The two main strategies of miRNA-based therapeutics are replacement and inhibition. The first strategy employs miRNA mimics, which imitate the structure and function of tumor-suppressive miRNAs that are downregulated in the tumor cell. The second strategy relies on miRNA inhibitors, which are antisense oligonucleotides (ASOs) designed to hybridize with oncomiRs, thereby inhibiting their activity [58].

A notable example of the current landscape of miRNA therapeutics is the Phase 1 study that evaluated a locked nucleic acid (LNA) inhibitor of the oncogenic miR-221. LNA-i-miR221 was administered to patients with advanced cancers and demonstrated a satisfactory safety profile, with an overall partial response (PR) and stable disease (SD) rate of 56.3%. This study (NCT04811898) supports further investigation of miRNAs as therapeutic targets [59].

Although no studies using miRNAs or miRNA-targeting molecules encapsulated in exosomes have yet been initiated, a recent example of a Phase 1 study—this time aimed at restoring the loss of function of miR-34a—is study NCT01829971. In this trial, MRX34, a miR-34a mimic encapsulated in liposomes, was administered to patients with advanced/refractory solid tumors and demonstrated encouraging results regarding safety and anticancer activity [60]. A more recent study in the preclinical setting has demonstrated in vitro induction of apoptosis and reduced migration and invasion in breast cancer cells using exosomes enriched with miR-34a derived from genetically modified dental pulp mesenchymal stem cells (DPSCs) [61].

## 5. Future Directions and Limitations

Through the advancement of liquid biopsy techniques, miRNAs can provide valuable information about the mechanisms that cancer cells rely on for their survival and proliferation, in a minimally invasive manner. Encapsulation within sEVs protects miRNAs from enzymatic degradation, resulting in greater stability than free-circulating RNA species, and the information obtained can be more accurately linked to the tumor status and may serve to monitor the disease with high specificity. A prospective evaluation of circulating EV-miRNAs may provide important information about the tumor’s dynamic state and reveal mechanistic dependencies that may render EV-miRNAs promising drug targets. However, as research endeavors to decipher the clinical significance of EV-miRNAs in diagnosis, prognosis, and therapeutic targeting in cancer, limitations—primarily of a technical nature—are encountered.

Although significant progress in understanding EV biology has been made, several important questions remain unresolved. sEV biogenesis, cargo composition, and cargo-loading mechanisms vary substantially among cell types and between physiological and pathological conditions. As our understanding of EV biology grows, the need to establish standardized isolation protocols that account for their complex biology becomes increasingly urgent [62]. This is because the sizes of different types of extracellular vesicles overlap. Specifically, regarding exosomes, MISEV2023 recommends that the term should be avoided unless there is certainty regarding the endosomal origin of the vesicles in question. This is because, given the overlapping size ranges, exosomes can easily be confounded with microvesicles, which are of plasma membrane origin [63].

A key reason why the characterization of extracellular vesicles has not yet been incorporated in clinical practice is that preanalytical and analytical factors still greatly influence their quantity and origin. These factors are difficult to control entirely, making standardization tricky and necessitating a consensus so that different studies can be comparable.

Studies demonstrating the effect of preanalytical factors on EV isolation and characterization underscore the need to emphasize standardization in EV-related research. Variables such as freeze–thaw cycles, centrifugation conditions, and storage time affect both the morphological characteristics and concentration of EVs as well as their protein and nucleic acid cargo [64]. The choice of anticoagulant also affects the quantity and origin of the vesicles detected in plasma. Citrate partially inhibits platelet activation that leads to the formation of platelet-derived EVs. Despite the fact that citrate has historically been the anticoagulant of choice for EV analysis, recent studies have shown that EDTA stabilizes the concentration of platelet-derived EVs more efficiently during blood collection and handling. Consequently, EDTA is increasingly recommended for studies focused on platelet-derived EVs, although the optimal anticoagulant may still depend on the intended downstream EV target [65].

Apart from preanalytical factors, detection and quantification methods are based on the heterogeneous physicochemical and biological characteristics of circulating particles. Examples of frequently used techniques for EV quantification include Nanoparticle Tracking Analysis (NTA) and Dynamic Light Scattering (DLS), which detect particles based on size and density by recording scattered light. Although sensitive, such techniques do not provide information regarding the origin of the vesicles detected, with the consequence that it is not possible to categorize them with certainty, a process that requires information about origin, as in the case of exosomes [66,67].

In the field of EV isolation from body fluids, primarily plasma, a wide variety of methods based on different principles are used. The choice of method should be determined by the objectives of the experiment. For instance, immunoaffinity-based capture (IAC) uses antibodies specific to target proteins on the surface of vesicles, resulting in the isolation of high-purity EVs but only of specific subsets depending on the antibodies selected [68]. For isolation in a biologically untargeted manner, methods based on physicochemical properties are preferred. Size-exclusion chromatography (SEC) is a microfluidics approach that separates EVs from debris by passing them through a microfluidics channel. In the case of SEC, enrichment of a broader set of EVs is achieved without preserving information about the cell type of origin [69]. The heterogeneity of isolation and quantification methods underscores the need for the standardization and validation of the available methods, as well as the rationale they will serve during experimental design.

In addition to the technical challenges discussed above, another significant challenge is the limited functional validation of EV-miRNA findings. While numerous studies, including high-throughput profiling approaches, have identified candidate miRNAs linked to cancer progression and therapeutic response, many of these findings remain largely correlational. Establishing causal relationships between specific EV-miRNAs and tumor behavior is hampered by the absence of reliable in vivo models and standardized functional assays. Additionally, the reproducibility and comparability of results across studies may be affected by variations in experimental design, including differences in sample sources, isolation techniques, and analytical platforms. Therefore, progress in understanding EV biology, the development of precise isolation methods, and the standardization of these methods are interrelated areas in which the field still has much to explore.

It is evident that EV analysis exhibits heterogeneity not only in the nature of circulating vesicles and the factors regulating their concentration and content, but also in the multitude of isolation methods. Consequently, in an effort to improve the robustness and reproducibility of these methods, and to ensure that findings are replicable across studies, the EV-TRACK knowledge base has been developed for reporting experimental metadata, enabling the interpretation and reproduction of the reported experiments. Such initiatives are crucial for maintaining transparency within the EV community and filtering out studies of questionable quality [70].

## 6. Conclusions

EV-miRNAs represent an important component of intercellular communication in health and disease, influencing multiple aspects of tumor biology, including proliferation, metastasis, angiogenesis, immune evasion, and therapy resistance. Their ability to modulate gene expression in recipient cells highlights their role as key mediators of tumor–tumor and tumor–microenvironment interactions. The increased stability of miRNAs within sEVs renders them promising candidates for minimally invasive liquid biopsy applications in cancer diagnosis, prognosis, and treatment monitoring. In addition, advances in miRNA-based therapeutics and the potential use of engineered sEVs as delivery vehicles further underscore their translational value in precision oncology. However, several challenges remain, including incomplete understanding of sEV biogenesis and miRNA sorting mechanisms, as well as the lack of standardized isolation and characterization methods for extracellular vesicles. Furthermore, the dynamic regulation of EV secretion and miRNA cargo composition in response to environmental and therapeutic pressures underscores the need for prospective longitudinal studies. Addressing these limitations through improved methodologies and large-scale clinical validation will be essential for the successful integration of EV-miRNAs into clinical practice and personalized cancer management. Future large-scale, multicenter clinical trials, such as NCT07243015 and NCT07225452 for gastric and pancreatic cancer, will determine the clinical utility of EV-miRNA panels in liquid biopsy. Their integration into routine screening protocols could transform the landscape of precision oncology.

## Figures and Tables

**Table 1:** Registered clinical trials in ClinicalTrials.gov investigating the utility of EV-miRNAs in cancer.

Clinical Trials	Population	Clinical Outcomes	Liquid Biopsy Method	Estimated Number of Participants	Study Phase/Evidence Tier
NCT07243015	Patients who underwent curative-intent gastrectomy for gastric adenocarcinoma, with available pre- and postoperative plasma samples	Recurrence-free survival (RFS) Overall survival (OS)	Small RNA sequencing RT-qPCR-based exo-miRNA quantification	500	Validation Phase (prospective evaluation of MRD signature)
NCT07225452	Individuals diagnosed with intrahepatic cholangiocarcinoma and control participants (non-cancer or benign biliary disease) with available pre-treatment plasma samples	Sensitivity Specificity Diagnostic accuracy (AUC)	Small RNA sequencing RT-qPCR validation of selected miRNAs	500	Discovery and Validation Phase (diagnostic biomarker development)
NCT07224802	Patients with histologically confirmed pancreatic ductal adenocarcinoma (PDAC) who have undergone curative-intent pancreatectomy with available preoperative plasma samples	Specificity Sensitivity Area under the receiver operating characteristic curve (AUC)	Small RNA sequencing RT-qPCR validation	400	Validation Phase (multicenter machine learning-based validation)
NCT07224737	Histologically confirmed intrahepatic cholangiocarcinoma (ICC) at clinical stage I–III, treatment with curative-intent hepatectomy and availability of a preoperative plasma or serum sample	Recurrence-free survival Overall survival	Small RNA sequencing RT-qPCR validation	250	Discovery and Validation Phase (machine learning-based predictive modeling)
NCT07224724	Patients diagnosed with colorectal liver metastases (CRLMs) originating from histologically confirmed colorectal adenocarcinoma at participating institutions	Sensitivity Specificity Accuracy	Small RNA sequencing RT-qPCR validation	500	Discovery and Validation Phase (machine learning-based model for occult EHM)
NCT06654622	The study will enroll patients with stage II-III colorectal cancer who have undergone curative surgery and require assessment for molecular residual disease to determine whether adjuvant chemotherapy is necessary	Tumor evaluation (recurrence) Overall survival (OS)	miRNA panel	200	Validation Phase (EMRATI score development for MRD-guided ACT)
NCT06490159	Patients needing second-line chemotherapy with unresectable or recurrent gastric cancer	Incidence of peripheral neuropathy	miRNA panel (RT-qPCR)	150	Discovery and Validation Phase (prediction of chemotherapy-induced toxicity)
NCT06381648	Individuals who were diagnosed with intrahepatic cholangiocarcinoma	Sensitivity Specificity Proportion of correct predictions among the total number of cases	miRNA panel (RT-qPCR)	190	Discovery and Validation Phase (preoperative prediction of lymph node metastasis)
NCT06342427	Two cohorts of individuals with and without gastric cancer (cases and controls, respectively)	Sensitivity Specificity Proportion of correct predictions among the total number of cases	miRNA panel	809	Discovery and Validation Phase (multi-modal cf/sEV-miRNA signature for early detection)
NCT06342414	Individuals who were diagnosed with either intrahepatic cholangiocarcinoma or hepatocellular carcinoma (case–case design for a differential diagnosis study)	Sensitivity Specificity Proportion of correct predictions among the total number of cases	Small RNA sequencing from exo-miRNA	400	Discovery and Validation Phase (machine learning-driven differential diagnosis between HCC and ICC)
NCT06277986	The research object is patients with confirmed gastric cancer; according to diagnostic criteria, patients are divided into a cachexia group and a non-cachexia group	BMI	Plasma-derived exo-miRNA	150	Discovery and Validation Phase (biomarkers for early cancer cachexia detection)
NCT05854030	Patients diagnosed with advanced lung squamous carcinoma by histopathology and treated with anti-PD-L1 combined with chemotherapy	Plasma exosomal miRNA level PD-L1 Imaging data of lesions Objective response rate	RNA sequencing	60	Discovery and Validation Phase (predictive signature for anti-PD-L1/chemotherapy response)
NCT04629079	The study will include patients who have been referred to the Lung Cancer Clinic and Multi- Disciplinary Team (MDT) at The Lister, Hertford County and New QEII Hospitals for investigation of suspected lung cancer	Describe the range of exosomal expression of P4HA1 Describe the range of expression of precursor microRNA in exosomes Develop a combined risk score	-	800	Clinical Evaluation Phase (prospective validation of combined CT/sEV-hypoxia risk score for lung cancer screening)
NCT04167722	Obese vs. lean patients	Determine differences in peri-prostatic adipose tissue Identification of exosomal small RNAs transferred between adipose tissue to prostate cancer cell lines Assess how exosomal small RNAs from lean vs. obese patients affect cancer regulation Attempt to replicate functional changes observed	Small RNA sequencing	100	Discovery and Translational Phase (investigation of adipocyte-derived sEV crosstalk in prostate cancer progression)
NCT03911999	For the non-prostate cancer group, there is no specific time limit for urine collection; for the prostate cancer group, urine will be collected prior to prostatectomy	To compare the differences in microRNA expression between non-prostate cancer subjects, pathologically insignificant and significant prostate cancer patients To assess the accuracy of selected microRNAs for the differentiation of patients with pathologically insignificant and significant prostate cancer after radical prostatectomy	Exosomal RNA (next generation sequencing)	180	Discovery and Validation Phase (urinary sEV-miRNA for differentiating significant vs. insignificant prostate cancer)
NCT03886571	Patients with an upcoming standard of care clinical and/or surgical event who meet criteria for study participation are identified by the treating physician and will be asked to participate in the study	Measuring cell-free and exosomal miRNA biomarkers using small RNA-Seq in matched tissue and plasma from patients with PDAC, PNs, pancreatitis and normal pancreas for early detection	Small RNA sequencing	100	Translational Infrastructure (large-scale biobanking for multi-analyte EV discovery and validation)
NCT02366494	Men with systemic disease (with biochemical relapse or metastatic disease)	Identify five most prevalent exosomal microRNAs that predict response to androgen deprivation therapy-based treatment Identify exosomal microRNAs that predict response to androgen deprivation therapy (ADT) from peripheral blood of prostate cancer patients with systemic disease Validate exosomal RNA markers that predict response to ADT by real-time RT-PCR	Exosomal RNA (next-generation sequencing) Validate exosomal RNA (RT-PCR)	42	Discovery and Validation Phase (NGS-based signatures for predicting duration of response to ADT)
NCT07226154	Patients diagnosed with resectable or borderline resectable pancreatic ductal adenocarcinoma (PDAC) who received neoadjuvant chemotherapy (FOLFIRINOX or Gemcitabine + Nab-paclitaxel) followed by surgery	Pathological response rate Recurrence-free survival (RFS) Overall survival (OS) Radiologic response rate	Small RNA sequencing RT-PqCR validation	200	Discovery and Validation Phase (predictive miRNA panel for neoadjuvant chemotherapy response in PDAC)

## Data Availability

No new data were created or analyzed in this study. Data sharing is not applicable to this article.

## Supplementary Materials

The following supporting information can be downloaded at: https://www.mdpi.com/article/10.3390/cancers18121903/s1, Table S1: Registered clinical trials (ClinicalTrials.gov) investigating the role of sEV-derived miRNA in various cancer types.

## Abbreviations

(sEVs) small extracellular vesicles, (EV-miRNAs) extracellular vesicles-associated micro-RNA, (miRNAs) microRNAs, (miR) microRNAs, (MVBs) multivesicular bodies, (ILVs) intraluminal vesicles, (ESCRT complex) endosomal sorting complex required for transport, (RBPs) RNA Binding Proteins, (EMT) epithelial–mesenchymal transition, (BrCa) breast cancer, (LUAD) lung adenocarcinoma, (OS) osteosarcoma, (CAFs) cancer-associated fibroblasts, (VEGF) Vascular endothelial growth factor, (OSCC) oral squamous cell carcinoma, (NPC) nasopharyngeal carcinoma, (3′-UTR) untranslated region-3, (TME) tumor microenvironment, (CAFs) cancer-associated fibroblasts, (TP53INP1) tumor protein 53-induced nuclear protein 1, (BMSCs) bone marrow stem cells, (TNBC) triple-negative breast cancer, (NSCLC) non-small cell lung cancer, (TAMs) tumor-associated macrophages, (GC) gastric cancer, (HCC) hepatocellular carcinoma, (AR) acute tumor response, (LACC) locally advanced cervical cancer, (CCRT) concurrent chemoradiotherapy, (ASOs) antisense oligonucleotides, (LNA) locked nucleic acid, (SD) stable disease, (DPSCs) dental pulp mesenchymal stem cells.
