# Supplementary material for: Small Extracellular Vesicle-Associated microRNA in Cancer: Biology and Applications in Translational Research and Precision Oncology
Source: Cancers (Basel). 2026 Jun 11;18(12):1903. doi: 10.3390/cancers18121903 (PMC13297223; doi:10.3390/cancers18121903)
Supplement: Supplementary file 1 [file cancers-18-01903-s001.zip › cancers-4292134-supplementary.pdf]

Supplementary Table S1. Registered clinical trials (ClinicalTrials.gov) investigating the role of sEV-derived miRNA in various cancer types.

Clinical Trials	*Population*	*Clinical Outcomes*	*Liquid Biopsy Method*	*Status*	*Estimated Number of Participants*	*Sponsor*	*Study Phase / Evidence Tier*	*Clinical Utility (Diagnosti/ Prognostic/ Predictive)*	*Validation Status*
**NCT07243015**	Patients who underwent curative-intent gastrectomy for gastric adenocarcinoma, with available pre- and postoperative plasma samples	Recurrence-Free Survival (RFS),<br><br>Overall Survival (OS)	Small RNA sequencing<br><br>RT-qPCR-based exo-miRNA quantification	Recruiting	500	City of Hope Medical Center, USA	Validation Phase (Prospective evaluation of MRD signature)	Prognostic (primary), Predictive (intended/ secondary)	External validation (ongoing)
**NCT07225452**	Individuals diagnosed with intrahepatic cholangiocarcinoma and control participants (non-cancer or benign biliary disease) with available pre-treatment plasma samples	Sensitivity,<br><br>Specificity,<br><br>Diagnostic accuracy (AUC)	Small RNA sequencing<br><br>RT-qPCR validation of selected miRNAs	Completed	500	City of Hope Medical Center, USA	Discovery & Validation Phase (Diagnostic biomarker development)	Diagnostic (primary)	External validation (ongoing)
**NCT07224802**	Patients with histologically confirmed pancreatic ductal adenocarcinoma (PDAC) who have undergone curative-intent pancreatectomy with available	Specificity,<br><br>Sensitivity,<br><br>Area Under the Receiver Operating Characteristic Curve (AUC)	Small RNA sequencing<br><br>RT-qPCR validation	Completed	400	City of Hope Medical Center, USA	Validation Phase (Multi-center Machine Learning-based Validation)	Prognostic (primary), Predictive (intended)	External validation (ongoing)

	preoperative plasma samples								
**NCT07224737**	Histologically confirmed intrahepatic cholangiocarcinoma (ICC) at clinical stage I–III, treatment with curative-intent hepatectomy and availability of a preoperative plasma or serum sample	Recurrence<br><br>Free Survival,<br><br>Overall Survival	Small RNA sequencing<br><br>RT-qPCR validation	Completed	250	City of Hope Medical Center, USA	Discovery & Validation Phase (Machine Learning-based predictive modeling)	Prognostic (primary), Diagnostic (secondary), Predictive (intended)	External validation (ongoing)
**NCT07224724**	Patients diagnosed with colorectal liver metastases (CRLM) originating from histologically confirmed colorectal adenocarcinoma at participating institutions	Sensitivity,<br><br>Specificity,<br><br>Accuracy	Small RNA sequencing<br><br>RT-qPCR validation	Recruiting	500	City of Hope Medical Center, USA	"Discovery & Validation Phase (Machine Learning-based model for occult EHM)	Diagnostic (primary), Predictive (secondary/intended)	External validation (ongoing)
**NCT06654622**	The study will enroll patients with stage II-III colorectal cancer who have undergone curative surgery and require assessment for molecular residual disease to determine whether adjuvant chemotherapy is necessary	Tumor Evaluation (Recurrence),<br><br>Overall Survival (OS)	miRNA panel	Completed	200	City of Hope Medical Center, USA	Validation Phase (EMRATI score development for MRD-guided ACT)	Predictive (primary), Prognostic (secondary)	External validation (ongoing)
**NCT06490159**			miRNA panel	Recruiting	150				

	Patients need second-line chemotherapy with unresectable or recurrent Gastric cancer	Incidence of peripheral neuropathy	(RT-qPCR)			City of Hope Medical Center, USA	Discovery & Validation Phase (Prediction of chemotherapy-induced toxicity)	Predictive (primary)	External validation (ongoing)
**NCT06381648**	Individuals who were diagnosed with Intrahepatic cholangiocarcinoma	Sensitivity,<br><br>Specificity,<br><br>Proportion of correct predictions among the total number of cases	miRNA panel (RT-qPCR)	Completed	190	City of Hope Medical Center, USA	Discovery & Validation Phase (Preoperative prediction of lymph node metastasis)	Diagnostic (primary), Predictive (secondary)	External validation (ongoing)
**NCT06342427**	Two cohorts of individuals with and without gastric cancer (cases and controls, respectively)	Sensitivity,<br><br>Specificity,<br><br>Proportion of correct predictions among the total cases	miRNA panel	Completed	809	City of Hope Medical Center, USA	Discovery & Validation Phase (Multi-modal cf/sEV-miRNA signature for early detection)	Diagnostic (primary), Prognostic (secondary)	External validation (ongoing)
**NCT06342414**	Individuals who were diagnosed with either intrahepatic cholangiocarcinoma or hepatocellular carcinoma (case-case design for a	Sensitivity,<br><br>Specificity,<br><br>Proportion of correct predictions among the total number of cases	Small RNA sequencing from exo-miRNA	Recruiting	400	City of Hope Medical Center, USA	Discovery & Validation Phase (Machine learning-driven differential diagnosis between HCC and ICC)	Diagnostic (primary)	External validation (ongoing)

	differential diagnosis study)								
**NCT06277986**	The research object is patients with confirmed gastric cancer.According to diagnostic criteria, patients are divided into cachexia group and non-cachexia group.	BMI	Plasma-derived exo-miRNA	Unknown status	150	Xijing Hospital, China	Discovery & Validation Phase (Biomarkers for early cancer cachexia detection)	Diagnostic (primary)	External validation (ongoing)
**NCT05854030**	Patients diagnosed as advanced lung squamous carcinoma by histopathology and be treated with anti-PD-L1 combined with chemotherapy	Plasma exosomal miRNA level,<br><br>PD-L1,<br><br>Imaging data of lesions,<br><br>Objective response rate	RNA sequencing	Unknown status	60	Tianjin Medical University Cancer Institute and Hospital, China	Discovery & Validation Phase (Predictive signature for anti-PD-L1/Chemotherapy response)	Predictive (primary), Prognostic (secondary)	External validation (ongoing)
**NCT04629079**	The study will include patients who have been referred to the Lung Cancer Clinic and Multi-Disciplinary Team	Describe the range of exosomal expression of P4HA1	---------	Unknown status	800	King's College London, UK	Clinical Evaluation Phase (Prospective validation of combined CT/sEV-hypoxia risk score for	Diagnostic (primary), Prognostic (secondary)	External validation (ongoing)

	(MDT) at The Lister, Hertford County and New QEII Hospitals for investigation of suspected lung cancer.	Describe the range of expression of precursor microRNA in exosomes<br><br>Develop a combined risk score					lung cancer screening)		
**NCT04167722**	Obese vs lean patients	Determine differences of peri-prostatic adipose tissue,<br><br>Identification of exosomal small RNAs transferred between adipose tissue to prostate cancer cells lines,<br><br>Assess how exosomal small RNAs from lean vs obese patients affect cancer regulation,<br><br>Attempt to replicate functional changes observed	Small RNA sequencing	Unknown status	100	Imperial College London	Discovery & Translational Phase (Investigation of adipocyte-derived sEV crosstalk in prostate cancer progression)	Preclinical / Translational	Discovery (no clinical validation cohort)

**NCT03911999**	For non-prostate cancer group, there is no specific time limit for urine collection. For prostate cancer group, urine will be collected prior to prostatectomy.	To compare the differences in microRNA expression between non-prostate cancer subjects, pathologically insignificant and significant prostate cancer patients,<br><br>To assess the accuracy of selected microRNAs for the differentiation of patients with pathologically insignificant and significant prostate cancer after radical prostatectomy	Exosomal RNA (next generation sequencing)	Completed	180	Chinese University of Hong Kong, Chine	Discovery & Validation Phase (Urinary sEV-miRNA for differentiating significant vs. insignificant prostate cancer)	Prognostic (primary), Diagnostic (secondary)	External validation (ongoing)
**NCT03886571**	Patients with an upcoming standard of	Measuring cell-free and exosomal-miRNA	Small RNA sequencing	Unknown status	100	Hoag Memorial	Translational Infrastructure (Large-	Preclinical / Translational	Discovery

	care clinical and/or surgical event who meet criteria for study participation are identified by the treating physician and will be asked to participate in the study.	biomarkers using small RNA-Seq in matched tissue and plasma from patients with PDAC, PNs, pancreatitis and normal pancreas for early detection.				Hospital Presbyteria n, USA	scale biobanking for multi-analyte EV discovery and validation)			
**NCT02366494**	Men with systemic disease (with biochemical relpase or metastaic disease)	Identify five most prevalent exosomal microRNAs that predict response to androgen deprivation therapy based treatment.<br><br>Identify exosomal micro RNAs that predict response to Androgen deprivation therapy (ADT) from peripheral blood of prostate cancer patients with systemic disease.<br><br>Validate exosomal RNA markers that predict response to ADT by real-time RT-PCR.	Exosomal RNA (next-generation sequencing)<br><br>Validate exosomal RNA (RT-PCR)	Completed	42	Medical College of Wisconsin, USA	Discovery & Validation Phase (NGS-based signatures for predicting duration of response to ADT)	Predictive (primary), Prognostic (secondary)	External validation (ongoing)	

**NCT07226154**	Patients diagnosed with resectable or borderline resectable pancreatic ductal adenocarcinoma (PDAC) who received neoadjuvant chemotherapy (FOLFIRINOX or Gemcitabine + Nab-paclitaxel) followed by surgery.	Pathological Response Rate<br><br>Recurrence-Free Survival (RFS)<br><br>Overall Survival (OS)<br><br>Radiologic Response Rate	Small RNA sequencing<br><br>RT-PqCR validation	Recruiting	200	City of Hope Medical Center, USA	"Discovery & Validation Phase (Predictive miRNA panel for neoadjuvant chemotherapy response in PDAC)	Predictive (primary), Prognostic (secondary)	External validation (ongoing)	
